# Bortezomib Rescues Ovariectomy-Induced Bone Loss via SMURF-Mediated Ubiquitination Pathway

**DOI:** 10.1155/2021/9661200

**Published:** 2021-12-31

**Authors:** Yuepeng Fang, Yang Liu, Zhijian Zhao, Yingjie Lu, Xu Shen, Tianfeng Zhu, Mingzhuang Hou, Fan He, Huilin Yang, Yijian Zhang, Qin Shi, Xuesong Zhu

**Affiliations:** ^1^Department of Orthopaedics, The First Affiliated Hospital of Soochow University, Soochow University, Suzhou 215006, China; ^2^Orthopaedic Institute, Medical College, Soochow University, Suzhou 215007, China; ^3^Jinan Central Hospital, Cheeloo College of Medicine, Shandong University, No. 105, Jiefang Road, Jinan, Shandong 250013, China

## Abstract

A balance between bone formation by osteoblasts and bone resorption by osteoclasts is necessary to maintain bone health and homeostasis. As a cancer of plasma cells, multiple myeloma (MM) is accompanied with rapid bone loss and fragility fracture. Bortezomib has been used as a first-line for treating MM for decades. Recently, the potential protection of bortezomib on osteoporosis (OP) is reported; however, the specific mechanism involving bortezomib-mediated antiosteoporotic effect is undetermined. In the present study, we assessed the effects of *in vitro* bortezomib treatment on osteogenesis and osteoclastogenesis and the protective effect on bone loss in ovariectomized (OVX) mice. Our results indicated that bortezomib treatment increased osteogenic differentiation of MC3T3-E1 cells as evidenced by increased levels of matrix mineralization and osteoblast-specific markers. In bortezomib-treated bone marrow monocytes (BMMs), osteoclast differentiation was suppressed, substantiated by downregulated tartrate-resistant acid phosphatase- (TRAP-) positive multinucleated cells, areas of actin rings, pit formation, and osteoclast-specific genes. Mechanistically, bortezomib exerted a protective effect against OP through the Smad ubiquitination regulatory factor- (SMURF-) mediated ubiquitination pathway. Furthermore, *in vivo* intraperitoneal injection of bortezomib attenuated the bone microarchitecture in OVX mice. Accordingly, our findings corroborated that bortezomib might have future applications in the treatment of postmenopausal OP.

## 1. Introduction

Osteoporosis (OP) is a progressive metabolic disease manifested as degeneration of bone structure and impaired bone mass [1]. Approximately 71.8 million people over 50 years suffer from OP in China, a 25-fold higher prevalence than in the United Kingdom [2]. Severe OP is accompanied by increased bone fragility, leading to increased risks of osteoporotic vertebral compression fracture (OVCF) and hip fracture [3], associated with considerable morbidity and mortality. Mechanically, although OP can be divided into two subtypes: estrogen-deficient OP and senile OP based on the etiology, the collective driving factor is disrupted homeostasis between bone formation and bone resorption [4]. An osteoblast/osteocyte-induced osteogenesis shift toward overactivated osteoclast-mediated osteoclastogenesis can lead to excessive bone loss and microarchitectural impairment [5]. Therefore, targeting the regulation of bone metabolism is essential for reversing the progression of OP.

Bortezomib is a boronic acid dipeptide first reported as an antineoplastic agent for multiple myeloma (MM) [6]. Bortezomib exerts anticancer effects during oncologic therapy by inhibiting proteasome [7], inducing apoptosis and cell cycle arrest. Recently, bortezomib has been used in the treatment of non-hematologic malignancies, including degenerative musculoskeletal diseases (osteoarthritis, OA) [8], rheumatoid arthritis (RA) [9], and titanium particle-induced osteolysis [10]. Accumulating data indicate that during MM treatment, bortezomib also affects bone metabolism [11]. Suominen et al. reported that *in vivo* combination treatment of bortezomib and radium-223 alleviated myeloma-induced bone destruction in the syngeneic 5TGM1 MM mice model [12]. Furthermore, in mice models of ovariectomy-induced OP, intraperitoneal injection of bortezomib delayed the bone loss by inhibiting bone resorption [13]. However, the underlying mechanisms by which bortezomib modulates bone homeostasis are far from being known.

As a posttranslational modification, ubiquitination has been documented in multiple biological processes [14]. A classical ubiquitin-proteasome system (UPS) consists of three main factors: ubiquitin-activating enzyme (E1), conjugating enzyme (E2), and protein ligase (E3) [15]. Interestingly, bone metabolism can be modulated by the ubiquitination of transforming growth factor- (TGF-) *β* and bone morphogenetic protein (BMP) signaling pathways via Smad ubiquitination regulatory factors (SMURFs) [16]. SMURF1 promoted myogenic differentiation but blocked BMP2-mediated osteogenic conversion by activating the ubiquitin degradation of Smad5 in mouse C2C12 myoblast cells [17]. Moreover, a small skeletal phenotype and activated bone resorption were found in *Smurf2*^−/−^ mice with increased ubiquitin levels of Smad1/5 [18]. Intriguingly, Wang et al. demonstrated that bortezomib prevented prostate cancer oncogenesis and bone metastasis by modulation of ubiquitination regulatory factors SMURF1 and SMURF2 [19]. Nevertheless, the mechanisms involving bortezomib-induced ubiquitination in osteoporosis are not elucidated.

Our study was aimed at exploring the effect of *in vitro* bortezomib treatment on osteoblast and osteoclast differentiation. The protection of bortezomib on preventing bone loss in ovariectomized- (OVX-) impaired mice was also determined. In addition, the potential mechanisms underlying the bortezomib-induced antiosteoporotic effect were explored by targeting SMURF-mediated ubiquitination.

## 2. Materials and Methods

### 2.1. Cell Culture and Bortezomib Treatment

The osteoblastic cell line MC3T3-E1 was purchased from the Cell Bank of the Chinese Academy of Sciences (Shanghai, China). MC3T3-E1 cells were cultured in a medium containing alpha minimum essential medium (*α*-MEM) (Thermo Fisher Scientific, Waltham, MA, USA), 10% fetal bovine serum (FBS), and 1% penicillin and streptomycin at 37°C with 5% CO_2_. For osteogenic differentiation, MC3T3-E1 cells were seeded in 12-well plates and cultured with an osteogenic differentiation medium (*α*-MEM supplemented with 10% FBS, 50 *μ*g/mL ascorbic acid, 10 mM *β*-glycerophosphate, and 100 nM dexamethasone). As previously described, bone marrow monocytes (BMMs) were isolated from C57BL/6J mice [20]. Briefly, bone marrow cells were flushed from the tibias and femurs of mice using a syringe. The marrow cells were incubated with red blood cell lysis buffer (Beyotime, Nantong, China) to remove red blood cells (RBC). Cells were cultured in 10 cm dishes with a standard growth medium (*α*-MEM supplemented with 10% FBS and 1% penicillin and streptomycin) at 37°C with 5% CO_2_. The suspended cells were collected and treated with 100 ng/mL macrophage-colony stimulating factor (M-CSF) (R&D Systems, Minnesota, USA). For the induction of osteoclast differentiation, BMMs were seeded in 12-well plates and incubated with *α*-MEM with 50 ng/mL receptor activator of nuclear factor-*κ*B ligand (RANKL) and 30 ng/mL M-CSF (R&D Systems). To determine the *in vitro* effect of bortezomib (Sigma-Aldrich) on both osteoblast and osteoclast differentiation, cells were divided into three groups: negative control (NC) group, induced differentiation group, and 1 nM of bortezomib-treated group.

### 2.2. Alkaline Phosphatase Staining

After 7 days of osteogenic differentiation, MC3T3-E1 cells were washed with phosphate-buffered saline (PBS) and fixed with 4% paraformaldehyde. The cells were stained with BCIP/NBT Alkaline Phosphatase (ALP) Color Development Kit (Beyotime) for 30 min at room temperature. After washing with PBS, images were captured using an Olympus IX51 microscope (Olympus Corporation, Tokyo, Japan). ALP activity was measured using an ALP assay kit (Beyotime) and normalized to the protein concentration.

### 2.3. Alizarin Red S Staining

After 14 days of osteogenic differentiation, MC3T3-E1 cells were fixed with 4% paraformaldehyde for 15 min. The cells were then incubated with 40 mM Alizarin Red S solution (Sigma-Aldrich, St. Louis, MO, USA) for 30 min at room temperature. Digit images were observed using an inverted microscope. The stain was extracted with 5% perchloric acid solution, and the matrix mineralization level was quantified using a spectrophotometer (BioTek).

### 2.4. F-Actin Staining

BMMs were seeded on a 24-well plate and treated with 50 ng/mL RANKL and 30 ng/mL M-CSF to induce osteoclasts. To determine the effect of bortezomib, cells were treated with 1 nM bortezomib and the culture media were changed every other day. After 5 days of osteoclastogenic differentiation, BMMs were washed with PBS and fixed with 4% paraformaldehyde. The cells were incubated with 0.1% Triton X-100 for 20 min and stained with CytoPainter Phalloidin-iFluor 488 Reagent (Abcam, Cambridge, MA, USA) for 1 h. Cell nuclei were counterstained using a 4′,6-diamidino-2-phenylindole (DAPI) for 4 min. Immunofluorescence images of F-actin rings were observed with an inverted fluorescence microscope. The number of F-action rings was measured with an ImageJ software.

### 2.5. Pit Formation

BMMs were seeded on 96-well plates coated with the inorganic bone biomaterial surface (Corning Life Sciences, NY, USA). After 6 days of osteoclast differentiation, cells were removed with a 2% hypochlorite solution for 10 min at room temperature. After washing with PBS, the resorption pits were observed under a microscope.

### 2.6. TRAP Staining

BMMs were fixed using a 4% paraformaldehyde solution. The cells were stained with an acetate-buffered solution containing naphthol AS-BI phosphate and tartrate (Sigma-Aldrich) at 37°C for 1 h. TRAP-positive multinucleated cells were counted and photographed using an Olympus IX51 microscope.

### 2.7. RT-PCR

Total RNA was extracted using a TRIzol reagent, and 1 *μ*g of RNA was used to synthesize cDNA using a cDNA Synthesis Kit. Quantitative real-time RT-PCR was performed using an SYBR Green Supermix kit. Transcript levels of *Alp*, *Col1a1*, *Runx2*, *Sp7*, *Bglap*, *Smurf1*, *Smurf2*, *Trap*, *Ctsk*, *Mmp9*, and *Nfatc1*, with *Gapdh*, were used as internal controls. The expression levels of mRNAs were calculated according to a *Δ*Ct (2^−*ΔΔ*Ct^) method. The primer sequences are shown in Table 1.

### 2.8. Western Blot

Cellular proteins were extracted from osteoclast or osteoblast using a cell lysis buffer (Beyotime) supplemented with proteinase inhibitors (Thermo Fisher Scientific) on ice for 1 h. The extracted protein was subjected to 10% sodium dodecyl sulfate-polyacrylamide gel (SDS-PAGE) followed by transferring electrophoretically onto a nitrocellulose membrane (Beyotime). The membranes were blocked and then incubated with primary antibodies against OPG (Abcam, ab73400), RUNX2 (Cell Signaling Technology, 12556S), OSX (ab209487), OCN (ab13420), SMURF1 (2174S), SMURF2 (12024S), P-SMAD1/5/9 (13820S), CTSK (ab37259), TRAP (ab191406), NFATC1 (8032S), MMP9 (13667S), P-P65 (Abclonal, AP0124), P-ERK (AP1120), and *β*-actin (3700S). Subsequently, membranes were incubated with the secondary antibodies for 1 h at room temperature. Bound antibodies were imaged on a VersaDocTM imaging system (Bio-Rad). After normalization against *β*-actin as an internal standard, the relative expression levels of proteins were calculated with an ImageJ Software.

### 2.9. Bioinformatic Prediction

The GSE156508 dataset containing osteoblast transcriptome from six controls and six osteoporotic patients was downloaded from the National Center for Biotechnology Information (NCBI) search database and Gene Expression Omnibus (GEO) database. Screening of differentially expressed genes (DEGs) and generation of volcano plots and heat maps were performed using R software. Protein and protein interaction (PPI) was conducted using an online analyzing tool STRING (https://string-db.org/). Gene Ontology (GO) and Kyoto Encyclopedia of Genes and Genome (KEGG) analyses were performed to explore the comprehensive biological process (BP), cellular component (CC), molecular function (MF), and signaling pathways.

### 2.10. OVX-Induced Bone Loss

Animal experiments were handled according to the protocols of the Ethics Committee of Soochow University. Eight-week-old female mice (C57BL/6J) were provided by the Experimental Animal Center of Soochow University. A total of 24 mice were randomly allocated into three groups (8 per group). After anesthesia with sodium pentobarbital, Group A mice were subjected to a sham operation, while Group B and C mice underwent bilateral ovariectomy. After surgery, mice were injected with penicillin intramuscularly for three days to avoid infection.

### 2.11. Administration of Bortezomib

Bortezomib was dissolved in dimethylsulfoxide (DSMO) (Sigma-Aldrich) and then diluted in saline for subsequent injection. One week after surgery, Groups A and B received an intraperitoneal injection of 0.9% saline, while Group C received an intraperitoneal injection of 0.5 mg/kg bortezomib. All injections were performed twice a week for six weeks. Mice were anesthetized with isoflurane and then euthanized using the cervical dislocation method eight weeks after surgery, and bilateral femurs were collected for the following analyses.

### 2.12. Microcomputed Tomography (*μ*CT) Analysis

The bone mass of distal femurs was evaluated using a *μ*CT system with a high resolution (16 *μ*m) at 60 kV (380 *μ*A). Data reconstruction was performed using NRecon v1.6 software. Regions of interest (ROI) were drawn 500 *μ*m from the end of the epiphyseal growth plate to points 1 mm along the cortical bone. The bone microarchitectural parameters, including bone mineral density (BMD), the bone volume ratio (BV/TV), bone surface/volume ratio (BS/BV), and trabecular number (Tb.N), were calculated.

### 2.13. Histology and Immunohistochemistry (IHC)

Femurs were fixed in formalin and then decalcified in Ethylenediaminetetraacetic Acid (EDTA) buffer (Yuanye) for four weeks. Bones were then dehydrated with 50%, 70%, 80%, and 90% ethanol and embedded in paraffin (Thermo Fisher Scientific). Longitudinal sections at 5 *μ*m thickness were cut and stained with hematoxylin and eosin (H&E) and TRAP staining. Osteoblast areas were evaluated with H&E staining, and osteoclast numbers were counted in TRAP-stained sections using ImageJ software. For immunohistochemical staining, the slides were retrieved using testicular hyaluronidase for 45 minutes. After blocking with 5% goat serum, the slides were incubated with anti-SMURF, anti-SMURF2, anti-OCN (23418-1-AP), and anti-p-SMAD1/5/9 primary antibodies at 4°C overnight. Then, the slides were incubated with a second antibody for 2 h. As previously described, the staining was performed using a 3,3′-Diaminobenzidine (DAB) method [21]. Digit images were observed under a bright-field microscope.

### 2.14. Statistical Analysis

Data are presented as the mean ± standard deviation (SD). Statistical analyses were conducted using an independent two-tailed Student's *t*-test for two groups or one-way Analysis of Variance (ANOVA) with Tukey's post hoc test for multiple groups using a GraphPad Prism 8. A probability value of *p* less than 0.05 was considered statistically significant.

## 3. Results

### 3.1. *In Vitro* Bortezomib Treatment Enhances the Osteogenesis of MC3T3-E1 Cells

**Figure 1:**
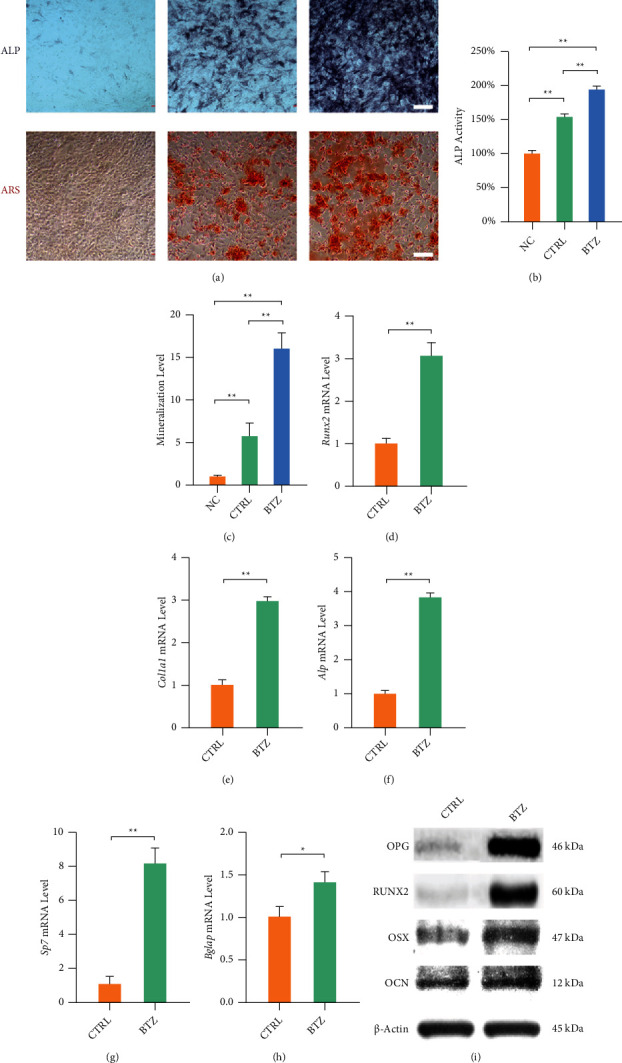
The effect of bortezomib on the osteogenic differentiation of MC3T3-E1 cells. (a) Representative images of mineralized extracellular matrix stained by Alkaline Phosphatase and Alizarin Red S staining. Scale bar = 200 *μ*m. (b) Quantification of ALP activity in bortezomib-treated MC3T3-E1 cells. (c) Quantification of stained mineral layers in bortezomib-treated MC3T3-E1 cells. The values were normalized to the level of the NC group. (d–h) The mRNA levels of osteoblast-specific marker genes, including *Runx2*, *Col1a1*, *Alp, Sp7*, and *Bglap* were quantified with real-time RT-PCR using GAPDH as the normalization. (i) The protein levels of osteoblast-specific marker enzymes including OPG, RUNX2, OSX, and OCN were determined using Western blot assays. Values represent mean ± SD of three replicas for ALP activity and ARS staining assays, three replicas for RT-PCR experiments and three replicas for Western blot assays, respectively. Statistically significant differences are indicated by ∗ where *p* < 0.05 or ∗∗ where *p* < 0.01 between the indicated groups.

After 7 days of osteogenic induction, the number of ALP-positive cells was higher in the bortezomib-treated group than in the free-treated cells (Figure 1(a)). Quantitative analysis indicated that bortezomib treatment increased the ALP activity in MC3T3-E1 cells by 26.5% (Figure 1(b)). After 2 weeks of osteogenic differentiation, Alizarin Red S staining revealed that bortezomib treatment promoted calcium deposits in the matrix of MC3T3-E1 cells. The level of calcium deposits was increased by 1.8-folds in the bortezomib-treated group (Figure 1(c)). RT-PCR revealed that, after 2 weeks of osteogenic induction, bortezomib treatment upregulated the mRNA expressions of *Runx2* by 207.1%, *Col1a1* by 197.6%, *Alp* by 282.9%, *Sp7* by 718.0%, and *Bglap* by 41.6% in comparison with the free-treated group (Figures 1(d)–1(h)). Western blot confirmed that the protein levels of osteogenesis were consistent with their transcript levels (Figure 1(i) and Fig. S1A–C).

### 3.2. *In Vitro* Bortezomib Treatment Suppresses Osteoclast Differentiation of BMMs

**Figure 2:**
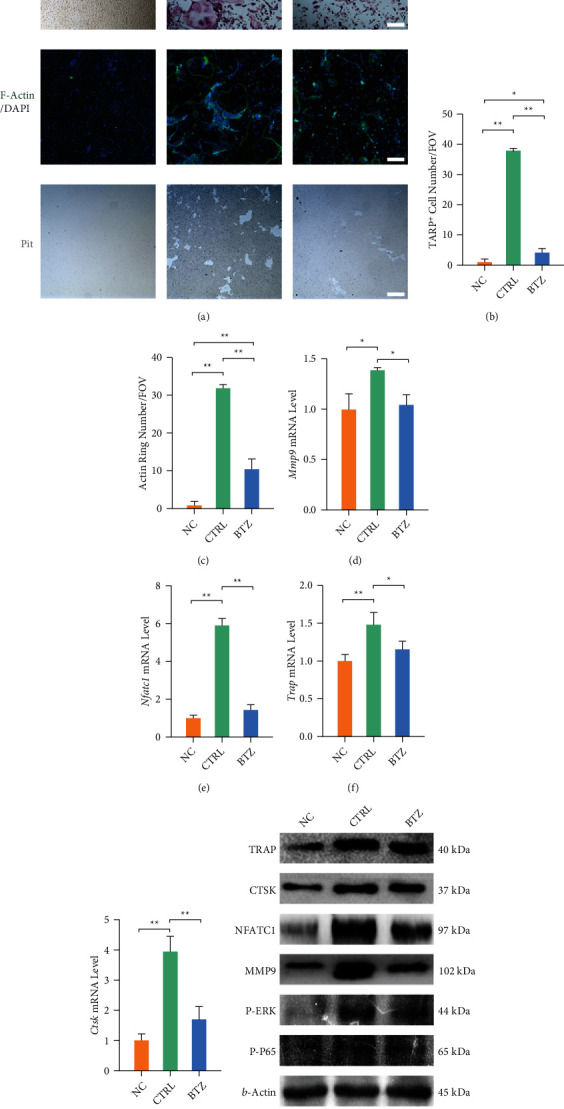
The effect of bortezomib on the osteoclastogenic differentiation of BMM cells. (a) Representative images of multinucleated osteoclasts stained by TRAP, F-actin rings, and pit formation. Scale bar = 200 *μ*m. (b) Quantification of the cell number of TRAP-positive osteoclasts in randomly chosen fields of view (FOV) of the bortezomib-treated BMM cells. (c) Quantification of the number of F-actin rings in randomly chosen FOV of the bortezomib-treated BMM cells. (d–g) The mRNA levels of osteoclast-specific marker genes, including *Trap*, *Ctsk*, *Nfatc1*, and *Mmp9* were quantified with real-time RT-PCR using GAPDH as the normalization. (h) The protein levels of osteoblast-specific marker enzymes were determined using Western blot assays. Values represent mean ± SD of three replicas for TRAP and F-actin staining assays, three replicas for RT-PCR experiments and three replicas for Western blot assays, respectively. Statistically significant differences are indicated by ∗ where *p* < 0.05 or ∗∗ where *p* < 0.01 between the indicated groups.

The TRAP staining showed that bortezomib treatment inhibited osteoclastogenesis in BMMs (Figure 2(a)). Quantitative results indicated the inhibition of the number and the area of the TRAP-positive cells in the bortezomib-treated group (Figure 2(b) and Fig. S2A). The administration of bortezomib suppressed the formation of F-actin rings as observed by the decreased number and area (Figure 2(c) and Fig. S2B). Similarly, osteoclast-induced bone resorption on the bone slices was restrained with the treatment of bortezomib (Fig. S2C). Furthermore, after five days of osteoclastogenic induction, bortezomib treatment decreased the transcript levels of *Trap* by 28.2%, *Ctsk* by 135.2%, *Nfatc1* by 307.3%, and *Mmp9* by 34.2% (Figures 2(d)–2(g)). Western blot confirmed that *in vitro* treatment with bortezomib inhibited the protein levels of osteoclast differentiation markers, as well as restrained the ERK-MAPK and NF-*κ*B signaling pathways (Figure 2(h) and Fig. S2D–I).

### 3.3. Bioinformatic Analysis between Control and Osteoporotic Osteoblast

**Figure 3:**
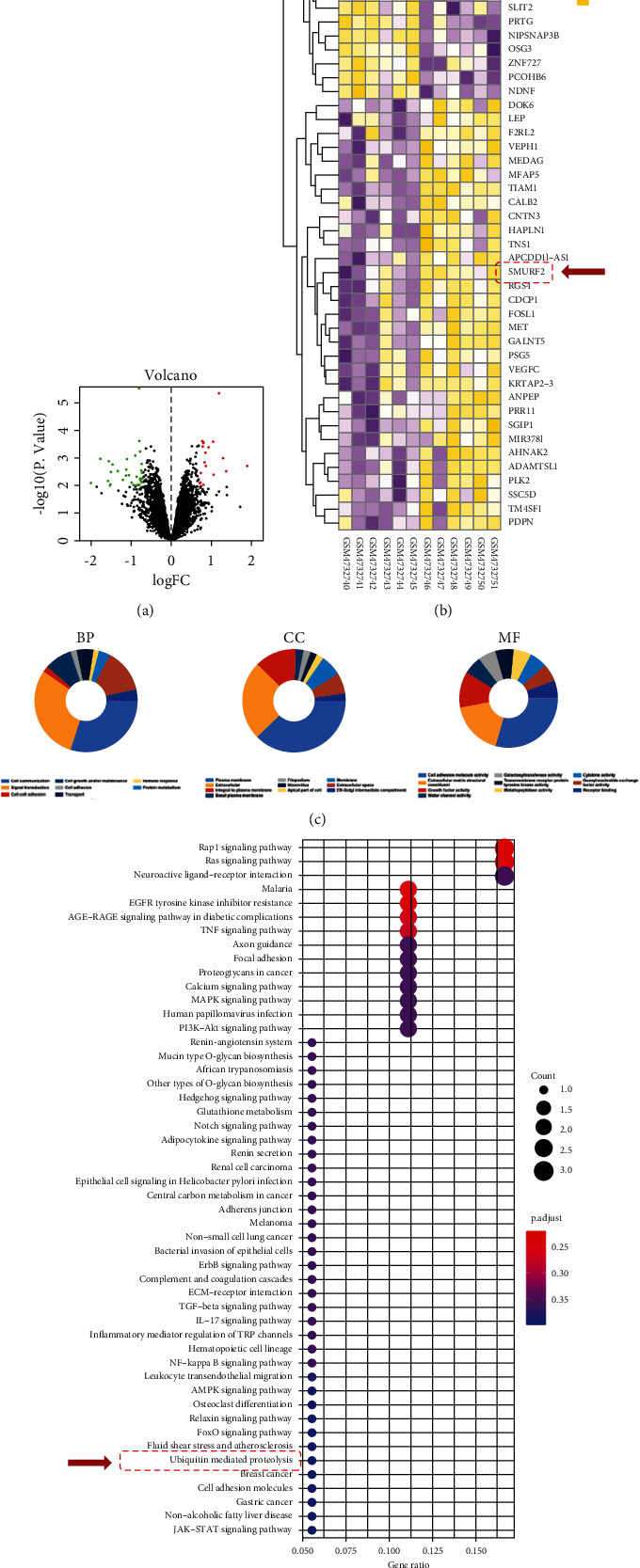
Bioinformatic analysis between the normal and OP patients. (a) Volcano map for the differentially expressed genes between the normal and OP patients. (b) Heat map for the differentially expressed genes between the normal and OP patients. (c) GO enrichment analysis for target genes according to biological process (BP), cellular component (CC), and molecular function (MF). (d) KEGG enrichment analysis for targets genes. Size of the dots (gene count) represents the number of genes. Color of the dots represents the *p* value.

We performed a bioinformatic analysis on the GEO dataset GSE156508 consisting of osteoblast transcriptome data from diseased samples and normal controls to explore the mechanisms underlying bortezomib-induced bone protection. A total of 48 DEGs, of which 17 were upregulated, and 31 were downregulated, were screened from the control and osteoporosis groups (Figure 3(a)). A heat map revealed that the transcript levels of *Smurf2* were decreased by 81.2%, *Rgs4* was decreased by 98.2%, and *Hey2* was increased by 77.6-folds in the OP group (Figure 3(b)). GO enrichment showed that cell communication, plasma membrane, and cell adhesion molecule activity were key biological behaviors in OP progression (Figure 3(c)). Pathway enrichment analysis revealed that multiple pathways, including mitogen-activated protein kinase (MAPK) signaling pathway (hsa04010), osteoclast differentiation (hsa04380), and ubiquitin-mediated proteolysis (hsa04120), participated in the development of OP (Figure 3(d)).

### 3.4. Administration of Bortezomib Delays OVX-Induced Bone Loss

**Figure 4:**
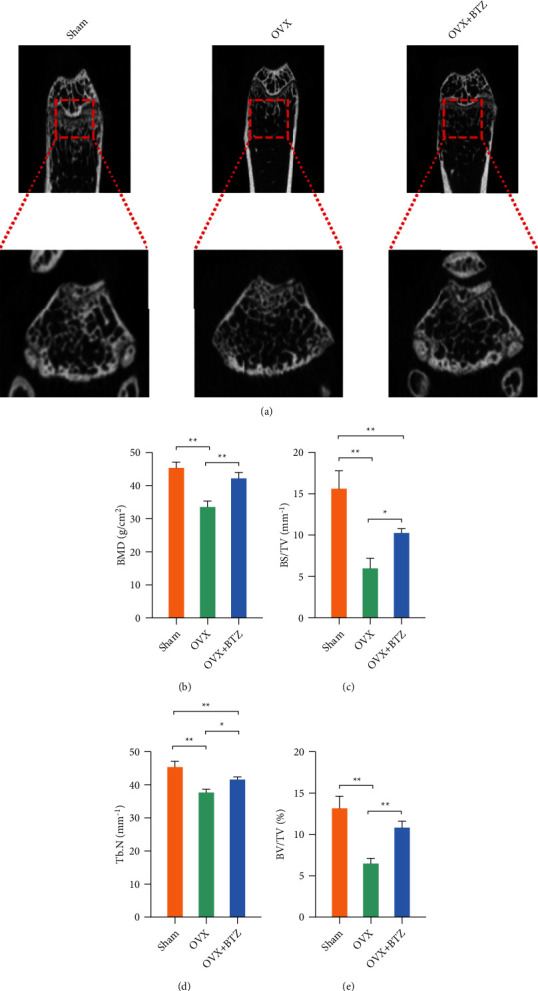
The effect of bortezomib on the radiographic bone loss in OVX mice. (a) Representative images of micro-CT reconstruction for coronal and cross-sectional plane of mice femurs. (b) Quantification of bone microstructure parameters including bone mineral density (BMD), the bone volume ratio (BV/TV, %), bone surface/volume ratio (BS/BV, mm^−1^), and trabecular number (Tb.N, mm^−1^). Values represent mean ± SD of six samples in each group (*n* = 6) in micro-CT assays. Statistically significant differences are indicated by ∗ where *p* < 0.05 or ∗∗ where *p* < 0.01 between the indicated groups.

**Figure 5:**
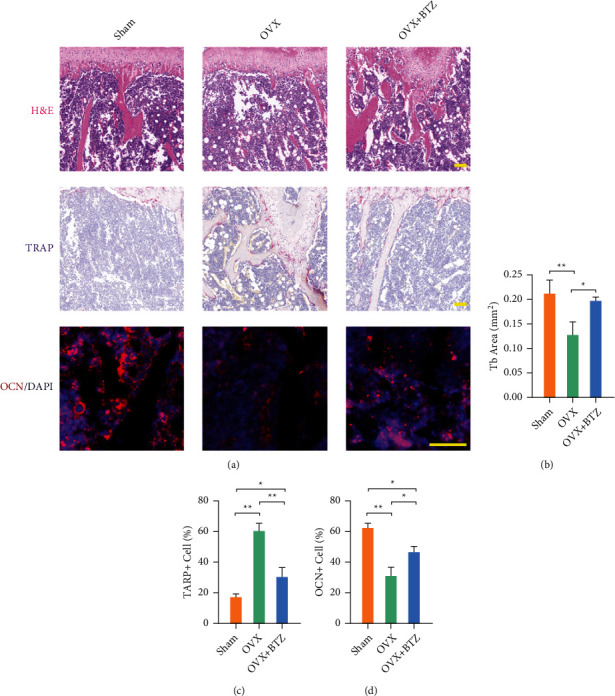
The effect of bortezomib on the histological bone loss in OVX mice. (a) Representative histological images of rat femurs stained by hematoxylin and eosin (H&E), tartrate-resistant acid phosphatase (TRAP), and OCN immunofluorescence staining. Scale bar = 100 *μ*m. (b) Quantification of bone microstructure parameters including Tb area. (c, d) The percentage of the number of TRAP- or OCN-positive cells. Values represent mean ± SD of six samples in each group (*n* = 6) in histological staining assays. Statistically significant differences are indicated by ∗ where *p* < 0.05 or ∗∗ where *p* < 0.01 between the indicated groups.

To determine the antiosteoporotic effect of bortezomib on estrogen-deficient OP, OVX mice received intraperitoneal injection of bortezomib. Eight weeks after bortezomib treatment, *μ*CT analysis showed restored bone loss in the bortezomib-treated group (Figure 4(a)). Quantitatively, administration of bortezomib increased BMD by 25.5%, BV/TV by 66.4%, BS/TV by 72.3%, and Tb.N by 10.5% (Figures 4(b)–4(e)). Furthermore, histomorphological staining corroborated the bone protection of bortezomib treatment *in vivo* (Figure 5(a)). The H&E staining confirmed that the Tb area was increased by 54.4% in the bortezomib-treated OVX mice (Figure 5(b)). *In vivo* TRAP staining revealed that bortezomib treatment reduced the TRAP-positive cells by 50.7% in OVX mice (Figure 5(c)). The percentage of OCN-positive cells was increased by 26.6% with bortezomib treatment compared with the OVX mice (Figure 5(d)).

### 3.5. Bortezomib Exerts Antiosteoporotic Effects via Suppressing SMURF-Mediated SMAD Ubiquitination

**Figure 6:**
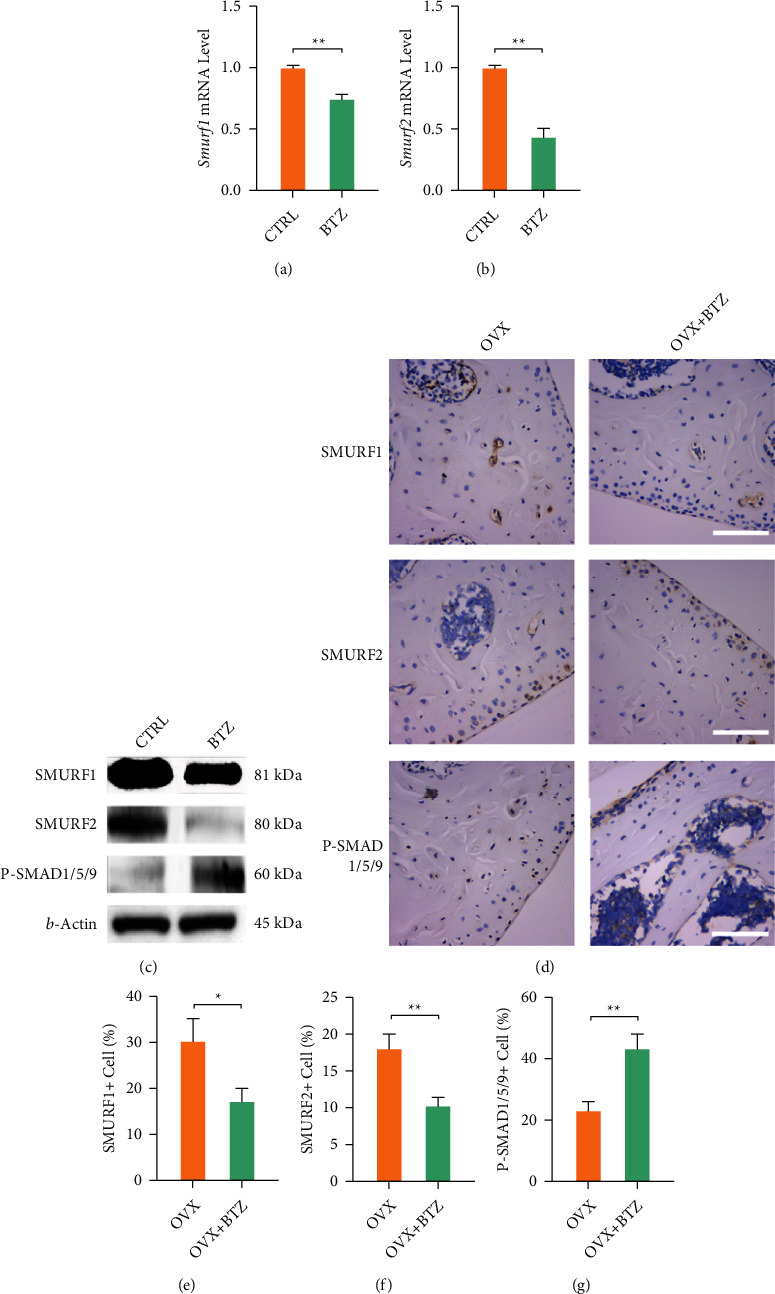
The involvement of SMURF1/2 in the bortezomib-mediated antiosteoporotic effect. (a, b) The mRNA levels of *smurf1* and *smurf2* genes were quantified with real-time RT-PCR using GAPDH as the normalization. (c) The protein levels of SMURF enzymes and SMAD pathway were determined using Western blot assays. (d) Representative immunohistology images stained by SMURF1, SMURF2, and p-SMAD1/5/9. (e–g) Quantification of the cell number of SMURF1-, SMURF2-, or P-SMAD-1/5/9-positive cells. Values represent mean ± SD of six samples in each group (*n* = 6) in histological staining assays. Statistically significant differences are indicated by ∗ where *p* < 0.05 or ∗∗ where *p* < 0.01 between the indicated groups.

Based on the above bioinformatic results, we further investigated the role of SMURF-mediated ubiquitination in the antiosteoporotic effect of bortezomib. RT-PCR showed that bortezomib treatment downregulated the mRNA levels of *Smurf1* by 25.9% and *Smurf2* by 56.8% (Figures 6(a) and 6(b)). Western blot revealed that the protein levels of SMURF1 and SMURF2 were decreased in the bortezomib-treated group. However, bortezomib promoted a 1.4-fold activation of the p-SMAD1/5/9 pathway, as evidenced by the Western blot experiment (Figure 6(c) and Fig. S3A–C). Furthermore, the IHC experiments revealed the effects of *in vivo* administration of bortezomib on the SMURF-mediated ubiquitination pathway (Figure 6(d)). Bortezomib treatment inhibited the expressions of SMURF1 by 43.0% and SMURF2 by 44.0% in OVX mice (Figures 6(e) and 6(f)); however, the p-SMAD1/5/9 expression level was improved by 88.8% in bortezomib-treated mice (Figure 6(g)).

## 4. Discussion

Bone remodeling is a physiological process that integrates osteoblast-induced bone formation and osteoclastic-mediated bone resorption and maintains skeletal integrity and mineral homeostasis [22]. Under different circumstances, including estrogen deficiency or aging, bone marrow stem cells (BMMSCs) can exhibit lower osteogenic potential and enhanced adipogenesis and osteoclastogenesis [23]. Targeting the dynamic balance between osteoblast and osteoclast differentiation is potentially key to developing new OP treatment methods. Given that the bone not only acts as a rigid structure to support body motion but also acts as the primary site of hematopoiesis, commonly used antihematologic neoplasm agents may be potential antiosteoporotic candidates. Cumulative shreds of evidence indicate that MM-related bone disorder is characterized by an imbalance between bone formation and resorption and disturbance of the RANKL-RANK-osteoprotegerin (OPG) axis [24]. As an MM-targeted drug, bortezomib was first found in the late 20^th^ century and used clinically for over ten years [25]. Past studies suggested that bortezomib could potentially be used for OP treatment based on elevated serum bone-specific alkaline phosphatase levels after bortezomib treatment [26]. Further research provided *in vitro* and *in vivo* evidence that bortezomib promoted osteoblast differentiation in MM patients [27].

**Figure 7:**
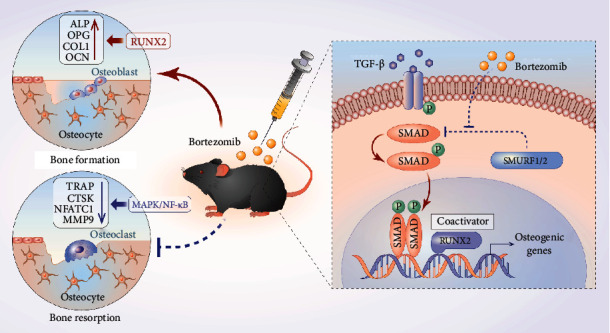
*In vivo* administration of bortezomib alleviates OVX-induced bone loss in mice. Bortezomib exerts a dual effect on promoting osteogenesis and suppressing osteoclastogenesis *in vitro*. Mechanically, bortezomib-mediated antiosteoporotic effect depends on the SMURF-related ubiquitination pathway.

Since bortezomib exhibited greater potential for treating OP, we analyzed its effects on osteogenesis and osteoblastogenesis (Figure 7). Accordingly, we found that 1 nM of bortezomib enhanced the osteogenic potential of MC3T3-E1 cells during the early (7 days) and late (14 days) induction periods. A previous study has reported a stimulating effect of *in vitro* bortezomib on osteoblast differentiation [28]. In our study, bortezomib upregulated the levels of osteoprotegerin (OPG) and runt-related transcription factor 2 (RUNX2), two genes involved in osteoblast differentiation. OPG reportedly acts as an inhibitor of osteoclast differentiation by disturbing the RANK/RNAKL activation pathway [29]. In this regard, OPG-knockout (KO) mice exhibited severe osteoporosis with over-activated osteoclast formation [30]. Moreover, RUNX2 has been reported to be a master modulator of osteoclast differentiation. Expression of RUNX2 has been associated with cranial suture closure and membranous bone morphogenesis at the embryonic stage [31]. Furthermore, postnatal global deletion of RUNX2 can result in bone loss and excessive bone marrow adiposity in mice bone tissues [32].

In addition to promoting osteogenesis, we revealed that the same concentration of bortezomib inhibited osteoclast differentiation in BMM osteoclast precursors. Consistent with the reduced number of TRAP-positive multinucleated osteoclasts, differentially expressed proteins (e.g., TRAP, MMP9, CTSK, and NFATc1) involved in osteoclastogenesis were downregulated by bortezomib. Moreover, 2 nM of bortezomib was reported to achieve similar findings in another study [13], corroborating that bortezomib can induce potent antiosteoclastogenesis effects. However, the mechanisms underlying bortezomib-mediated osteoclastogenesis inhibition are unknown and warrant further studies. Much controversy surrounds several aspects (e.g., dosage and drug-delivery methods) essential for the *in vivo* application of bortezomib in animal models. Although bortezomib has exhibited good clinical outcomes for MM treatment, it has been associated with several adverse events, e.g., peripheral neuropathy, cardiovascular toxicity, and skeletal muscle weakness [33]. Intraperitoneal injection of bortezomib for 30 days suppressed the growth and invasion of chondrosarcoma in BALB/c athymic nude mouse osteosarcoma model in a study by Bao et al. [34]. Using a similar *in vivo* drug delivery method, we found bortezomib exerted a protective effect on bone loss in C57BL/6J mice. Considering the adverse effects associated with bortezomib, including the potential risk for other organ systems, and determining which method of drug administration (local or systemic) may be more beneficial, Wang et al. performed intra-articular injections of bortezomib (0.5 mg/mL) in posttraumatic OA mice, which showed a decreased joint tissue damage and reduced inflammatory levels [35]. Interestingly, a novel tumor microenvironment-targeted nanoparticle loaded with bortezomib was designed to target the MM-associated endothelium and synchronize drug delivery to enhance clinical outcomes in MM and reduce the side effects [36]. Moreover, in another study, bortezomib-loaded nanomedicine assembled by pH-responsive properties showed minimal cytotoxicity at near-neutral pH and exerted an enhanced anticancer activity for metastatic bone tumors [37].

Herein, we performed bioinformatic analyses and sought to unravel the mechanisms underlying bortezomib modulation of osteoblast and osteoclast differentiation. SMURF-mediated ubiquitination pathway was found to be involved in the protective effect exerted by bortezomib against postmenopausal OP. Overexpression of SMURF2 was documented to delay fracture healing by modulating the ubiquitin level of the Wnt/*β*-catenin signaling pathway of *in vivo* femur fracture models [38]. Additionally, in an aging-induced intervertebral disc degeneration (IVDD) mice model, ectopic expression of SMURF2 accelerated proteoglycan loss and collagen fibrosis of nucleus pulposus (NP) [39]. Intriguingly, osteoblasts derived from OP patients decreased by 81.2% compared with control patients, which seems contrary to the negative role of SMURF2 in OP. We hypothesized that such contradiction might be attributed to confounding factors such as different OP stages and types. Intriguingly, beyond SMURF2, RGS4 and HEY2 were identified as the potential hub-genes. A latest study indicated that the expression of RGS4 was up-regulated during the osteogenic induction of adipose-derived stem cell (ADSC). Overexpression of LINC00370 sponge miR-222-3p to enhance RGS4-medicated bone protection [40]. Conversely, as a negative regulator, suppressing HEY2 with Notch2 antisense oligonucleotides (ASO) attenuated the cancellous osteopenia of Notch2*^tm1.1Ecan^* mice [41]. The potential amphoteric effects of bortezomib on RGS4 and HEY2 in OP will be explored in our further work.

Furthermore, according to our results, bortezomib-induced inhibition of SMURF was associated with increased expression of SMAD3. Xu et al. revealed that SMURF2 could modulate RANKL expression via blocking the interaction between SMAD3 and vitamin D receptor which was dependent on SMAD3 ubiquitination. Furthermore, SMURF2 can regulate osteoblast differentiation via ubiquitin/proteasome-mediated degradation of RUNX2 [42]. Therefore, the detailed mechanisms of the bortezomib-induced OP-antagonistic effect through SMURF warrants further exploration. Furthermore, multiple miRNAs, e.g., miR-615-3p [43], miR-30a-3p [44], miR-29b [45], and miR-324-5p [46], were found to be implicated in bortezomib-exerted anti-MM effect. The involvement of noncoding RNA will be further explored in our future work. Several limitations should be emphasized in this study. First, we only built an estrogen-deficient OP model; an aging-induced model was not studied due to time constraints. Second, further activation or knockdown experiments of SMURF are essential to improve the robustness of our findings and will be carried out in future studies. Meanwhile, the MC3T3-E1 cell line but not primary cells was studied in this experiment. In the subsequent experiment, we will investigate the salutary effects of bortezomib on rat-derived BMMSC and osteoporotic rat-derived BMMSC.

## 5. Conclusions

We demonstrated that bortezomib successfully enhanced the osteogenic potential and modulated osteoclastogenesis to regulate bone homeostasis *in vitro*. Furthermore, intraperitoneal administration of bortezomib improved the bone mineral density and microarchitecture in OVX-induced mice femurs *in vivo*. The bortezomib-mediated antiosteoporotic effect was achieved via the SMURF-mediated ubiquitination pathway. Accordingly, our findings substantiate that bortezomib may be a potential drug for treating osteoporosis.

## Figures and Tables

**Table 1:** Primers used for quantitative real-time RT-PCR.

Gene	Forward primer sequence (5′-3′)	Reverse primer sequence (5′-3′)
*Gapdh*	GGTGAAGGTCGGTGTGAACG	CTCGCTCCTGGAAGATGGTG
*Alp*	CCAACTCTTTTTGTGCCAGAGA	GGCTACATTGGTGTTGAGCTTTT
*Col1a1*	TCTCCACTCTTCTAGTTGGGAC	TTGGGTCATTTCCACATGC
*Runx2*	GTGACACCGTGTCAGCAAAG	GGAGCACAGGAACTTGGGAC
*Sp7*	GGAAAGGAGGCACAAAGAAGC	CCCCTTAGGCACTAGGAGC
*Bglap*	CTTGGTGCACACCTAGCAGA	CTCCCTCATGTGTTGTCCCT
*Trap*	GACCTCCAAGTTCTTATCCTCAC	ACTGATACCGTCTGTCATCCC
*Ctsk*	GACGCAGCGATGCTAACTAA	CCAGCACAGAGTCCACAACT
*Mmp9*	CTGGACAGCCAGACACTAAAG	CTCGCGGCAAGTCTTCAGAGAAG
*Nfatc1*	ACAGAGTTACCATTGGCAGGA	GCTTGAGATACCACCTTTCCG
*Smurf1*	GGTGGCACTGCACTCCTAGAAC	GCGCGGACCCAAACTACAAC
*Smurf2*	TGCACTAACAACCTGCCGAAAG	CTTGTCATTCCACAGCAAATCCA

## Data Availability

The data used to support the findings of this study are available from the corresponding authors upon request.

## Abbreviations

BTZ: Bortezomib
MM: Multiple myeloma
OP: Osteoporosis
OVX: Ovariectomized
BMMs: Bone marrow monocytes
TRAP: Tartrate-resistant acid phosphatase
SMURF: Smad ubiquitination regulatory factor
OVCF: Osteoporotic vertebral compression fracture
RA: Rheumatoid arthritis
UPS: Ubiquitin-proteasome system
TGF: Transforming growth factor
BMP: Bone morphogenetic protein
FBS: Fetal bovine serum
RBC: Red blood cells
M-CSF: Macrophage-colony stimulating factor
RANKL: Receptor activator of nuclear factor-*κ*B ligand
PBS: Phosphate-buffered saline
BMMSCs: Bone marrow stem cells
OPG: Osteoprotegerin
IVDD: Intervertebral disc degeneration
NP: Nucleus pulposus.

## Supplementary Materials

Supplementary Materials Supplementary Figure 1: quantification of the protein levels of osteogenic markers in bortezomib-treated MC3T3-E1 cells. Supplementary Figure 2: quantification of the bone resorption indicators and protein levels of osteoclastogenic markers in bortezomib-treated BMM cells. Supplementary Figure 3: quantification of the protein levels of ubiquitination markers in bortezomib-treated MC3T3-E1 cells.
