# Supplementary material for: Bortezomib Rescues Ovariectomy-Induced Bone Loss via SMURF-Mediated Ubiquitination Pathway
Source: Oxid Med Cell Longev. 2021 Dec 31;2021:9661200. doi: 10.1155/2021/9661200 (PMC8741347; doi:10.1155/2021/9661200)
Supplement: Supplementary Materials — Supplementary Figure 1: quantification of the protein levels of osteogenic markers in bortezomib-treated MC3T3-E1 cells. Supplementary Figure 2: quantification of the bone resorption indicators and protein levels of osteoclastogenic markers in bortezomib-treated BMM cells. Supplementary Figure 3: quantification of the protein levels of ubiquitination markers in bortezomib-treated MC3T3-E1 cells. [file 9661200.f1.docx]

**
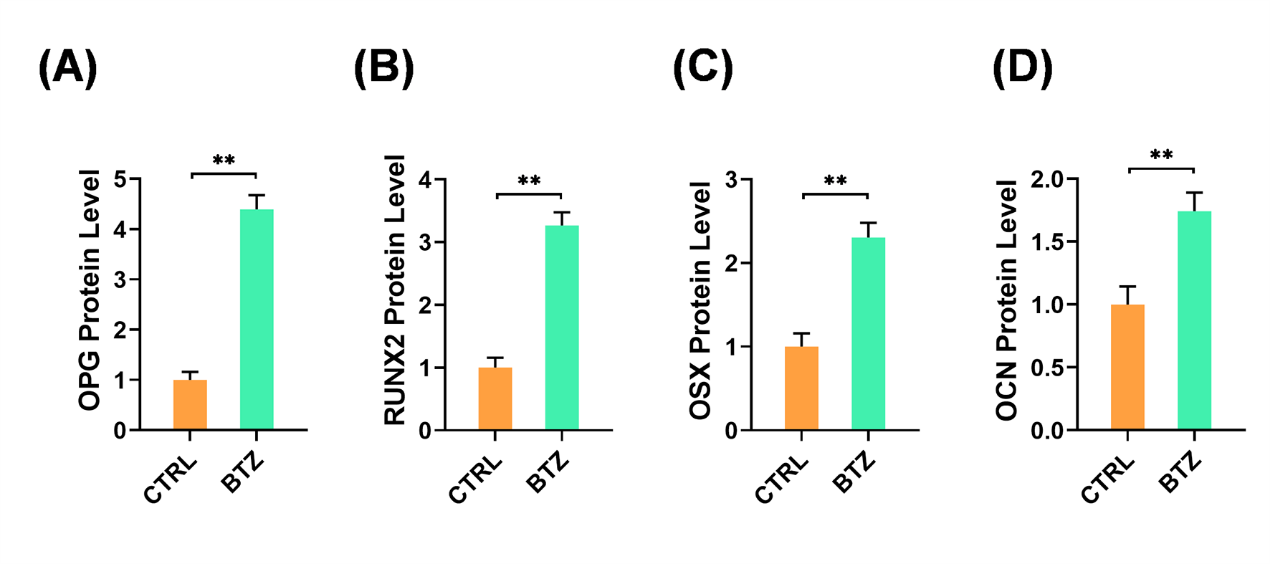
**

**Supplementary Figure 1.** Bortezomib treatment enhanced osteogenic potential of MC3T3-E1 cells. (A) Quantification of protein level of OPG in bortezomib-treated MC3T3-E1 cells. (B) Quantification of protein level of RUNX2 in bortezomib-treated MC3T3-E1 cells. (C) Quantification of protein level of OSX in bortezomib-treated MC3T3-E1 cells. (D) Quantification of protein level of OCN in bortezomib-treated MC3T3-E1 cells. Values represent mean ± SD of three replicas for Western blot assays, respectively. Statistically significant differences are indicated by * where *p* < 0.05 or ** where *p* < 0.01 between the indicated groups.


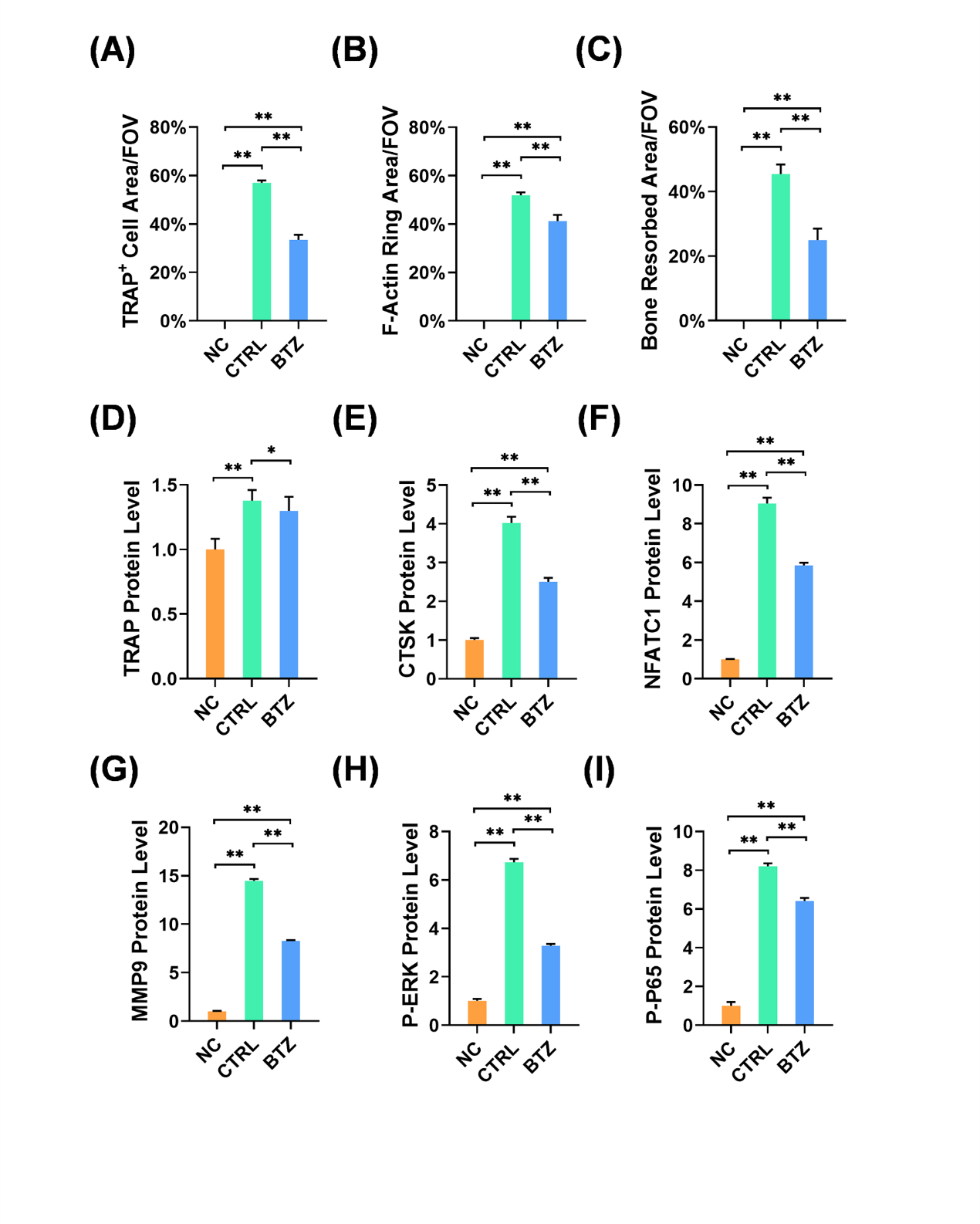


**Supplementary Figure 2.** Bortezomib treatment suppresses osteoclastogenic potential of BMM cells. (A) Quantification of the area of TRAP-positive osteoclasts in randomly chosen fields of view (FOV) of the bortezomib-treated BMM cells. (B) Quantification of the area of F-actin in randomly chosen fields of view (FOV) of the bortezomib-treated BMM cells. (C) Quantification of the osteoclast-induced resorption on bone slice in randomly chosen fields of view (FOV) of the bortezomib-treated BMM cells. (D) Quantification of protein level of TRAP in bortezomib-treated BMM cells. (E) Quantification of protein level of CTSK in bortezomib-treated BMM cells. (F) Quantification of protein level of NFATC1 in bortezomib-treated BMM cells. (G) Quantification of protein level of MMP9 in bortezomib-treated BMM cells. (H) Quantification of protein level of P-ERK in bortezomib-treated BMM cells. (I) Quantification of protein level of P-P65 in bortezomib-treated BMM cells. Values represent mean ± SD of three replicas for TRAP and F-actin staining assays, three replicas for bone resorption assays, and three replicas for Western blot assays, respectively. Statistically significant differences are indicated by * where *p* < 0.05 or ** where *p* < 0.01 between the indicated groups.

**
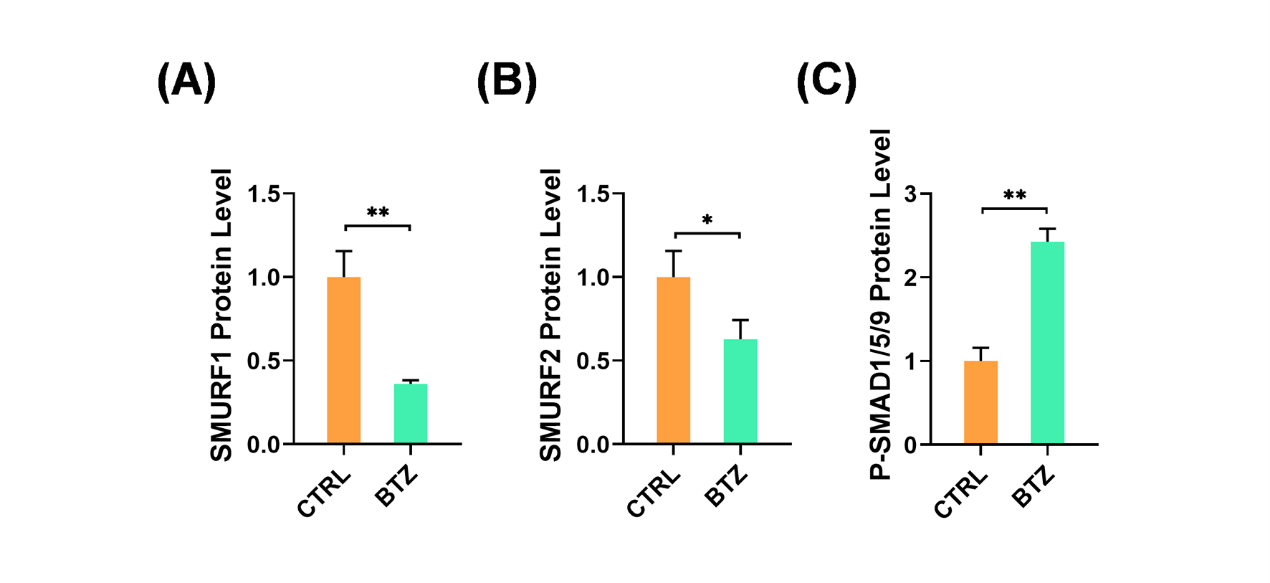
**

**Supplementary Figure 3.** Bortezomib treatment activates SMAD pathway by impeding SMURF expression. (A) Quantification of protein level of SMURF1 in bortezomib-treated MC3T3-E1 cells. (B) Quantification of protein level of SMURF2 in bortezomib-treated MC3T3-E1 cells. (C) Quantification of protein level of P-SMAD1/5/9 in bortezomib-treated MC3T3-E1 cells. Values represent mean ± SD of three replicas for Western blot assays, respectively. Statistically significant differences are indicated by * where *p* < 0.05 or ** where *p* < 0.01 between the indicated groups.
